# Economic Policy Uncertainty in China and Bitcoin Returns: Evidence From the COVID-19 Period

**DOI:** 10.3389/fpubh.2021.651051

**Published:** 2021-03-11

**Authors:** Tiejun Chen, Chi Keung Marco Lau, Sadaf Cheema, Chun Kwong Koo

**Affiliations:** ^1^Business School, Zhejiang University of Technology, Hangzhou, China; ^2^Business School, Teesside University, Middlesbrough, United Kingdom; ^3^Business School, The University of Huddersfield, Huddersfield, United Kingdom; ^4^Business School, Shanghai University, Shanghai, China

**Keywords:** COVID-19 pandemic, bitcoin, cryptocurrency markets, Chinese economy, EPU

## Abstract

This paper analyses the effects of the Chinese Economic Policy Uncertainty (CEPU) index on the daily returns of Bitcoin for the period from December 31, 2019 to May 20, 2020. Utilizing the Ordinary Least Squares (OLS) and the Generalized Quantile Regression (GQR) estimation techniques, the paper illustrates that the current CEPU has a positive impact on the returns of Bitcoin. However, the positive impact is statistically significant only at the higher quantiles of the current CEPU. It is concluded that Bitcoin can be used in hedging against policy uncertainties in China since significant rises in uncertainty leads to a higher return in Bitcoin.

**JEL Codes:** G32; G15; C22

## Introduction

The Global Financial Crisis (henceforth GFC) of 2008-09 destabilized the economic and financial stability of economies around the world and created high uncertainty about future economic security across the globe. During the GFC of 2008-09, Nakamoto (1) launched Bitcoin as an alternative to traditional currencies, which emerged as the most popular secure digital currency. However, as per its protocol design, the supply of Bitcoin is limited to 21 million. The occurrence of later financial crisis such as; the European Sovereign Debt Crisis of 2010-2013 and the Cypriot Banking Crisis of 2012-2013 further increased Bitcoin's popularity and established it as a “safe-haven” asset for investors (2–6).

As opposed to fiat currencies, cryptocurrencies are decentralized and act independently from government-regulated banking and other financial institutions. For instance, Corbet et al. (7) and Ji et al. (8) show that Bitcoin is independent of conventional assets and global financial system. Whereas, Demir et al. (9) state that cryptocurrencies provide solutions to the financial system's fragility and economic framework. Therefore, during times of economic and financial instability, investors withdraw their investment from traditional financial assets (like bonds, stocks etc.) to re-invest in Bitcoin to secure positive returns (9–12). Although initially introduced as an alternative to traditional currency, Bitcoin quickly emerged as a lucrative investment asset against conventional assets, so-termed Bitcoin as a “digital gold” (13).

Since Bitcoin behave independently from economic and financial developments (14); therefore, during times of extreme uncertainty or risk, Bitcoin offers significant diversification benefits for the investors. Bitcoin has the largest market capitalization and is considered as an alternative currency and medium of exchange. Bitcoin appears to be commodity money without gold, fiat money without state, and credit money without debt (15). The authors argued that Bitcoin provides a different opportunity for investments (16) as it embodies innovative technology and high security (17). Bitcoin has also created huge media attention in recent years, mainly due to its price fluctuations and potential for profit opportunities and transparency (18). Scholars have a different opinion about Bitcoin; some argue that Bitcoin is an efficient market (19, 20), others argue that Bitcoin is moving consistently toward efficiency (21). The researchers also argue that there are significant instability and bubbles in the Bitcoin. As a result, there is a speculative investment tendency related to Bitcoin (22). The year 2017 witnessed a drastic increase and decrease in Bitcoin demand, which brought scholars' attention to increasingly investigate Bitcoin's economic and financial determinants. It is the most widely used cryptocurrency, followed by Ethereum and Ripple (23).

In this paper, we re-examine the determinants of Bitcoin returns. For this purpose, we examine the effects of the index of economic policy uncertainty (EPU) in China on Bitcoin returns during the COVID-19 era since China has the largest mining pools for Bitcoin (24). Here, it is essential to know that the existing literature examining the impact of EPU on Bitcoin returns still inconclusive to present an undisputed argument with regards to Bitcoin hedging effectiveness against economic policy uncertainty capturing the COVID-19 period (25–28). Bitcoin returns can also affect households' consumption-savings trade-off in the COVID-19 period (29). Various studies assessing the role of economic policy uncertainty on different investment assets conclude that economic policy uncertainty has a positive influence not only on Bitcoin (9) but also on bonds (30) and commodities (31, 32). The empirical research has proved that Bitcoin remained resilient during times of uncertainty and stress, signifying its hedging capacity [see e.g., (6, 10–12)]. Whereas, Fang et al. (33), mainly reporting on the hedging effectiveness of Bitcoin, concludes that EPU has a relatively weak impact on Bitcoin's hedging performance, this finding contradicts that of (9). Since the literature on the impact of EPU on the hedging effectiveness of Bitcoin is still inconclusive; therefore, it is imperative to investigate this phenomenon further because such an inference is beneficial for the predictability of Bitcoin returns and improves investor's diversification and hedging decisions depending upon the level of economic policy uncertainty.

Therefore, the purpose of this study is to analyze the effects of the index of the Chinese Economic Policy Uncertainty (CEPU) on the daily returns of Bitcoin, considering the COVID-19-time period when uncertainty related to economic policy is higher. Furthermore, since the empirical literature examining the relationship between global economic policy uncertainty and Bitcoin returns is an under-researched area of study, therefore this study aims at investigating the role of Bitcoin to act as a hedging tool against economic policy uncertainty by considering the uncertainty caused by COVID-19 era in source country (China). Finally, we apply the OLS and the GQR estimation techniques to investigate (how Bitcoin returns are affected by the China EPU index) on the methodology side. This method allows us to see how extreme uncertainty affects Bitcoin returns and whether the CEPU can explain extreme Bitcoin returns. Considering the above factors, we relate our study to the existing empirical literature investigating the relationship and hedging effectiveness of Bitcoin against various variants of the EPU indices [see e.g., (9, 33–37)].

In the backdrop of the above information, the study continues as follows. Section Literature review provides the literature review. Section Data, model, and estimation procedure describes the data and the estimation procedure. Section Empirical Findings presents the empirical analysis and discussion of results. Section Conclusion provides concluding remarks.

## Literature Review

Bitcoin has become the most popular digital currency since its launch in 2008. The independent framework in which it operates without the regulations of central governments and traditional financial institutions has given Bitcoin much popularity. The cryptocurrency market technology depends on mass collaboration, and its decentralized system validates Bitcoin transactions to prevent fraud. The distributed ledger named Blockchain digitally stores all the transactions, thus ensuring security and integrity.

Recently, scholars have started to investigate the economic and financial traits of Bitcoin due to; Bitcoin's price volatility (38), bubble formation (7, 39, 40), mounting regulatory scrutiny and market manipulation (41), and speculative nature (40, 42). Scholars also investigated Bitcoin's use for money laundering purposes (43) or Ponzi schemes (44). Amongst financial scholars modeling Bitcoin volatility is a widely researched topic. Several studies used different models to analyze Bitcoin volatility persistence and spillovers [see e.g., (45–49)]. However, most of these studies have used GARCH-based models because of their ability to illustrate Bitcoin's conditional variance.

Similarly, another strand of literature relating to Bitcoin's international diversification and hedging ability is also increasingly explored by scholars. Several studies [see e.g., (2, 10, 42, 45, 50, 51)] investigated the role of Bitcoin as an effective hedging instrument, diversifier etc. For instance, using the multivariate quantile model, Wang et al. (5) examined the risk spillover effect from the U.S. EPU index to Bitcoin. It concluded that the effects are negligible/insignificant, affirming that Bitcoin can act as a safe-haven asset and a diversifier against EPU shock. Similarly, Wu et al. (37) compared Bitcoin and gold by investigating their hedge or safe-haven roles against EPU. The authors use the GARCH model and quantile regression with dummy variables, and the results indicate that the following results. First, both assets are unsuccessful at average to act as a reliable hedge or safe-haven against the EPU. Second, considering extreme market conditions, Bitcoin and Gold both act weakly against uncertainty shocks. Third, Bitcoin is more resilient against EPU shocks as compared to gold. These findings correspond to other studies [see e.g., (5, 9, 34)].

The extant empirical literature shows that the factors determining Bitcoin price are markedly different than those of conventional assets, for e.g., internet or google searches (52), the total number of unique Bitcoin transactions per day (53), information on media and google trends (54). Certain unique factors also determine Bitcoin price, e.g., energy prices (55), social sentiment (14, 56), technology (57), the ratio of exchange-traded volume and the hash rate (56). On investigating the potential drivers of Bitcoin (58) examined the determinants of Bitcoin returns by considering twenty-one potential variables that could drive Bitcoin returns. Using the least absolute shrinkage and selection operator (LASSO) regression, the authors conclude that search intensity, gold returns and policy uncertainty are the most significant drivers for Bitcoin returns.

Recently, a research topic that has become popular amongst scholars is the effect of uncertainty (i.e., uncertainty caused by geopolitical risk, trade policy uncertainty or economic policy uncertainty) on Bitcoin returns. Various empirical studies have examined the impact of uncertainty on Bitcoin returns to observe it's hedging effectiveness. For instance, Aysan et al. (59) examined the impact of geopolitical risks on Bitcoin returns and volatility. The authors used the GPR index developed by Caldara and Iacoviello (60) to measure global terrorism, wars, and tensions among states. Using the Bayesian graphical structural vector autoregressive model, Aysan et al. (59) find that GPR has a predictive power on price volatility and Bitcoin returns, therefore, signifying Bitcoin's ability to act as a useful hedging tool at times of higher global geopolitical risks.

Similarly, Gozgor et al. (36) explored the relationship between the trade policy uncertainty index and the Bitcoin returns of the United States. Using Wavelet Power Spectrum, Wavelet Coherency and Cross-Wavelet Techniques, the study results indicate that Bitcoin is positively related to the trade policy uncertainty index. However, at extreme periods of uncertainty, Bitcoin fails to serve as a hedge. In another study, Bouri et al. (34) investigate the relationship between global financial stress and Bitcoin returns. The authors used the global financial stress index as a proxy for global stress rather than using volatility indices (since the former better captures global stress). By employing a Copula-based approach to dependence and causality in the quantiles, the authors conclude that Bitcoin remained resilient during times of financial stress. Their findings correspond with other studies investigating Bitcoin returns' valuable role [see e.g., (2, 8, 51, 61)]. In their previous study, Bouri et al. (11) used volatility indices of 14 advanced and developing stock markets as a proxy for global uncertainty to examine whether Bitcoin acts as a hedging tool against global uncertainty. Using the standard OLS and quantile regression, the findings show that bitcoin is a useful hedging tool against uncertainty at higher quantiles and shorter frequency movements of Bitcoin returns.

It is a fact that the macroeconomic situation is a crucial factor that determines cryptocurrency returns (35). However, limited studies (9, 35, 37) have empirically examined if cryptocurrencies' returns are also affected by economic policy uncertainty. And this is the area where we intend to contribute to the literature. For example, Demir et al. (9) analyzed the U.S. EPU index's effect to predict Bitcoin returns. By applying the OLS and GQR estimations, the study results indicate that the EPU index has a predictive power for Bitcoin returns. The impact of EPU on Bitcoin returns is seen significantly positive at both lower and higher quantiles, which indicates that Bitcoin can be used as a hedging instrument against economic policy uncertainty. The authors conclude that EPU is very useful in predicting Bitcoin returns by arguing that investors lack confidence in traditional fiat currencies with an increase in economic policy uncertainty. Therefore, demand for cryptocurrencies increases.

In another study, Cheng and Yen (35) applied the predictive regression model, examining China's EPU index's impact on predicting major cryptocurrencies' returns (such as Bitcoin, Ethereum, Litecoin, and Ripple). The findings show that the China EPU index has significant predictive power for Bitcoin returns, whereas the EPU index of the U.S, Korea, and Japan has weak predictive power. The results also indicate that other than Bitcoin, the China EPU index does not possess predictive ability for other cryptocurrencies returns. Cheng and Yen (35) particularly highlight the U.S. EPU index results, which show no significant ability to predict Bitcoin returns and reported that their findings contradict Demir's et al. (9) findings. Cheng and Yen (35) argue that focusing on the long-run effect using monthly data and the U.S. EPU index possesses no predictive power for Bitcoin monthly returns. Similarly, Fang et al. (33) explored whether the volatility and hedging effectiveness of Bitcoin and other global assets is affected by economic policy uncertainty. The results indicate that Bitcoin's long-term volatility is significantly affected by EPU, whereas Bitcoin has weak hedging effectiveness against EPU.

The empirical studies (9, 11, 34, 59) proved that the association between Bitcoin and uncertainty change in upper quantiles implies that Bitcoin acts as a hedge only at times of higher uncertainty and risk. From the review of the above studies, we report that this study's results are similar and correspond to ones [e.g., (35)]. We conclude that higher uncertainty levels lead to positive returns on Bitcoin, which shows Bitcoin resilience and hedging capacity against the Chinese EPU index.

## Data, Model, and Estimation Procedure

### Data and Model

This empirical study uses daily frequency data and covers the period from December 31, 2019, to May 20, 2020. There are 142 observations; the sample period is purely dependent upon the data availability. The data for Bitcoin's logarithmic returns, which is treated as the dependent variable, is sourced from www.coindesk.com/price/. The daily China EPU index data, which is treated as an independent variable, is sourced from https://economicpolicyuncertaintyinchina.weebly.com/ constructed by Huang and Luk (62) following the methodology of Baker et al. (63). The pairwise correlation between the dependent and independent variable is 0.076. To investigate the return predictability of the China EPU index for Bitcoin returns, we estimate the following predictive model:

(1) $$\begin{array}{r} \Delta {\ln\left(BCP\right)}_{t}=\alpha_{0}+\alpha_{1}\Delta {\ln\left(CEPU\right)}_{t}+\varepsilon_{t} \end{array}$$

Where ln(*BCP*)_*t*_ represents the change rate of daily logarithmic returns of Bitcoin prices at time t; and ln(*CEPU*)_*t*_ represents the change rate of the EPU index in China at time t. ε_*t*_ represents the error term.

### Estimation Procedure

In this empirical investigation, we try to resolve different issues such as; misidentification of equations and implausible restrictions assumption (9, 34, 59). For example, Bouri et al. (34) predict the BRICS stock market returns using the VIX index to predict financial and macroeconomic variables. Therefore, following Bouri et al. (34), we assume the CEPU index as a potential predictor of the Bitcoin returns.

Moreover, in this empirical research, we use the Ordinary Least Squares (OLS) and the Generalized Quantile Regression (GQR) to model the quantile of Bitcoin returns (including various frequencies) as a function of the quantile of the CEPU index, which represents each point of their distributions. Additionally, the lagged effects of CEPU (up to 4 lags) on Bitcoin returns have also been examined in the empirical analysis.

## Empirical Findings

Table 1 presents the results of OLS and GQR estimations to understand the nature of this relationship. From the empirical results of OLS estimations, it is evident that the effects of CEPU and the lagged CEPU (up to 4 lags) are positive and statistically insignificant, which implies an increase in the EPU will not result in a jump in the Bitcoin returns or vice versa. However, at higher quantiles (see the quantiles from 0.75 to 0.95), the effects of the current CEPU on Bitcoin returns are still positive and statistically significant at the 5% level at least. The effects of the lagged CEPU on Bitcoin return is not statistically significant. Therefore, during higher quantiles, Bitcoin can serve as an effective hedging instrument against the Chinese policy uncertainty. Hence, investors are urged to consider China's daily economic policy uncertainty before investing in cryptocurrencies, enabling investors to predict Bitcoin returns better.

**Table 1:** Results of the OLS and the GQR estimations.

**Quantiles**	**CEPU**	**CEPU(−1)**	**CEPU(−2)**	**CEPU(−3)**
0.05	−0.166 (0.130)	0.662 (1.127)	−1.244 (1.121)	1.516 (1.374)
0.10	−0.363 (0.969)	0.673 (0.730)	−1.741 (0.930)	1.514 (0.974)
0.15	−1.103 (0.797)	0.597 (0.584)	−0.979 (0.910)	1.287 (0.899)
0.20	−0.923 (0.593)	0.390 (0.496)	−0.607 (0.667)	1.026 (0.956)
0.25	−0.816 (0.498)	0.663 (0.526)	−0.487 (0.630)	0.222 (0.548)
0.30	−0.134 (0.477)	0.240 (0.422)	−0.064 (0.586)	0.103 (0.524)
0.35	−0.074 (0.491)	0.052 (0.449)	−0.021 (0.613)	0.039 (0.538)
0.40	−0.003 (0.051)	0.013 (0.466)	−0.092 (0.616)	0.014 (0.538)
0.45	0.003 (0.053)	0.188 (0.477)	−0.435 (0.563)	0.045 (0.550)
0.50	0.113 (0.545)	0.303 (0.481)	−0.461 (0.561)	0.205 (0.568)
0.55	0.005 (0.051)	0.239 (0.494)	−0.767 (0.516)	0.291 (0.567)
0.60	0.026 (0.052)	−0.017 (0.545)	−0.796 (0.507)	0.264 (0.558)
0.65	0.168 (0.545)	−0.019 (0.567)	−0.776 (0.493)	0.601 (0.541)
0.70	0.287 (0.555)	−0.240 (0.596)	−1.330 (0.519)	1.189 (0.743)
0.75	0.776^**^ (0.358)	−0.304 (0.857)	−0.962 (0.637)	1.053 (0.708)
0.80	1.030^**^ (0.511)	−0.255 (0.896)	−0.660 (0.576)	0.879 (0.611)
0.85	1.029^**^ (0.463)	−0.007 (0.821)	−0.198 (0.543)	0.131 (0.512)
0.90	0.474^**^ (0.205)	−0.936 (1.158)	0.214 (0.565)	0.103 (0.484)
0.95	0.175^***^ (0.072)	−0.175 (0.102)	2.187 (1.642)	1.792 (1.508)
OLS	0.071 (0.076)	0.206 (0.776)	0.587 (0.807)	0.721 (0.820)

** p < 0.05 and

*** *p < 0.01. The Newey–West standard errors are in parentheses*.

**Figure 1:**
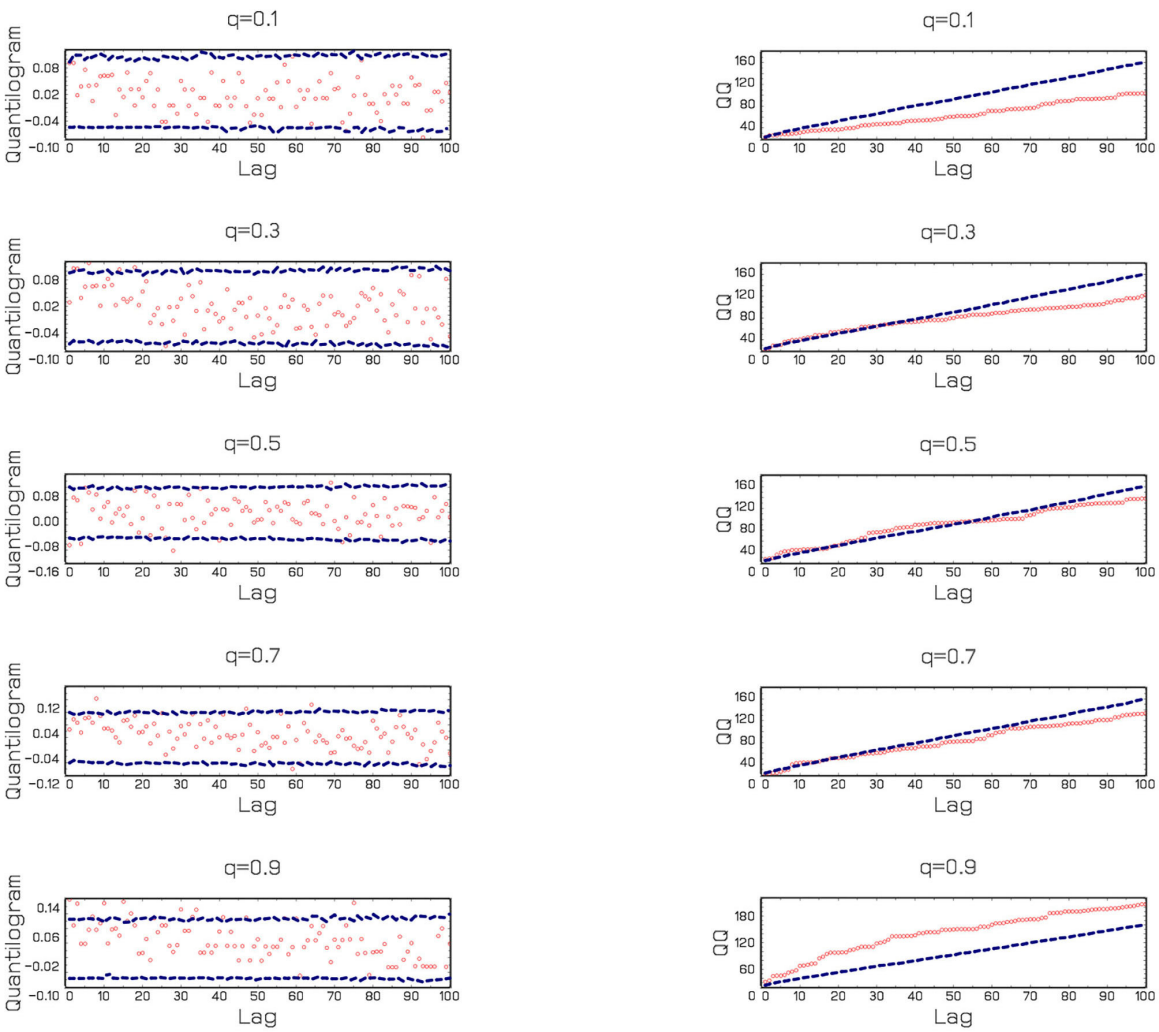
Results of the Q–Q Estimations of Sim and Zhou (64).

Figure 1 also provides additional results by running the Q-Q estimations procedure of Sim and Zhou (64). The findings indicate that the effects of CEPU on Bitcoin returns are positive in general. However, only quantile 0.9 exceeds the interval meaning that Bitcoin can hedge against the EPU in China at higher quantiles. This evidence shows that an event with extreme uncertainty is positively correlated to extreme Bitcoin returns.

As a robustness check, following the previous papers' models [e.g., (58, 65)], we have included control variables. However, we do not have a search intensity measure since the data are available at the Google Trends and Google Trends data do not capture China. We include gold returns at this stage, and our results are robust to include this measure (see Table 2).

**Table 2:** Results of the OLS and the GQR estimations (extended model with gold returns).

**Quantiles**	**CEPU**	**GOLD**
0.05	−0.056 (0.055)	−0.349 (0.302)
0.10	−0.050 (0.054)	−0.348 (0.251)
0.15	−0.035 (0.042)	−0.268 (0.214)
0.20	−0.024 (0.037)	−0.237 (0.184)
0.25	−0.018 (0.036)	−0.192 (0.159)
0.30	−0.014 (0.044)	−0.157 (0.160)
0.35	−0.003 (0.003)	−0.169 (0.167)
0.40	−0.001 (0.003)	−0.147 (0.173)
0.45	0.010 (0.029)	−0.090 (0.185)
0.50	0.024 (0.039)	−0.061 (0.208)
0.55	0.046 (0.042)	−0.042 (0.247)
0.60	0.144 (0.093)	−0.066 (0.264)
0.65	0.121 (0.144)	−0.054 (0.282)
0.70	0.154 (0.147)	−0.108 (0.281)
0.75	0.181^***^ (0.053)	−0.082 (0.301)
0.80	0.205^***^ (0.082)	−0.068 (0.414)
0.85	0.162^**^ (0.076)	−0.120 (0.401)
0.90	0.123^**^ (0.063)	−0.284 (0.421)
0.95	0.166^**^ (0.084)	−1.288 (0.803)
OLS	0.037 (0.051)	−0.150 (0.264)

** p < 0.05 and

*** *p < 0.01. The Newey–West standard errors are in parentheses*.

The empirical analysis findings indicate that Bitcoin can serve as an effective alternative hedging instrument against uncertainty. This evidence also provides potential implications for portfolio diversification and hedging (i.e., risk management). This study's results correspond to the findings of Bouri et al. (11) and Demir et al. (9), which conclude a positive relationship between economic policy uncertainty and Bitcoin returns. However, during extreme market conditions, Bitcoin can serve as a hedging tool against uncertainty. However, in times of normal market conditions, Bitcoin can be used for portfolio diversification.

Since the Bitcoin market is still at its early stage, the policymakers in China should consider that any uncertainty related to their economic policy could significantly affect the Bitcoin returns. This evidence is also established from this study's findings that the CEPU can effectively predict Bitcoin returns at higher quantiles. From this empirical analysis, it can be assumed that the Cryptocurrency market is quite vulnerable at the hands of uncertainty. Similarly, investors should weigh uncertainty related to economic policy and the existing natural uncertainty of cryptocurrencies before making investment decisions.

## Conclusion

This empirical investigation analyzed the relationship between China EPU (CEPU) index and Bitcoin returns from December 31, 2019 to May 20, 2020. We employ the OLS and the GQR estimations to investigate whether EPU in China has a predictive power on Bitcoin returns. It is observed that primarily, the Bitcoin returns are positively related to the CEPU at the higher quantiles. The results also indicate that the impact is significant and positive at the higher quantiles, which implies that Bitcoin can undoubtedly be used as a hedging instrument when uncertainty related to economic policy is higher. We suggest that the mechanism of the cryptocurrency market and its potential determinants should to understand better. Our findings are limited to the COVID-19 era and Bitcoin market. Given that we focus on the Chinese economic policy uncertainty, future research should explore the impacts of uncertainty on cryptocurrency markets (including altcoins) to disentangle economic uncertainty and COVID-19 related uncertainty measures in the post-COVID-19 era.

## Data Availability Statement

Publicly available datasets were analyzed in this study. This data can be found at: www.coindesk.com/price/; https://economicpolicyuncertaintyinchina.weebly.com.

## Author Contributions

CL, SC, and TC: formal analysis. TC and CK: funding acquisition and resources. CL: methodology. CL and TC: project administration. All authors contributed to the article and approved the submitted version.

## Conflict of Interest

The authors declare that the research was conducted in the absence of any commercial or financial relationships that could be construed as a potential conflict of interest.
